# Fully automated calculation of image-derived input function in simultaneous PET/MRI in a sheep model

**DOI:** 10.1186/s40658-016-0139-2

**Published:** 2016-02-13

**Authors:** Thies H. Jochimsen, Vilia Zeisig, Jessica Schulz, Peter Werner, Marianne Patt, Jörg Patt, Antje Y. Dreyer, Johannes Boltze, Henryk Barthel, Osama Sabri, Bernhard Sattler

**Affiliations:** Department of Nuclear Medicine, Leipzig University Hospital, Liebigstr. 18, Leipzig, Germany; Max Planck Institute for Human Cognitive and Brain Sciences, Stephanstr. 1a, Leipzig, D-04103 Germany; Fraunhofer Institute of Cell Therapy and Immunology, Perlickstr. 1, Leipzig, D-04103 Germany; Translational Centre for Regenerative Medicine, University Leipzig, Philipp-Rosenthal-Str. 55, Leipzig, D-04103 Germany; Fraunhofer Research Institution of Marine Biotechnology and Institute for Medical and Marine Biotechnology, University of Lübeck, Lübeck, Germany

**Keywords:** PET/MRI, Arterial input function, Image-derived input function, Angiography

## Abstract

**Background:**

Obtaining the arterial input function (AIF) from image data in dynamic positron emission tomography (PET) examinations is a non-invasive alternative to arterial blood sampling. In simultaneous PET/magnetic resonance imaging (PET/MRI), high-resolution MRI angiographies can be used to define major arteries for correction of partial-volume effects (PVE) and point spread function (PSF) response in the PET data. The present study describes a fully automated method to obtain the image-derived input function (IDIF) in PET/MRI. Results are compared to those obtained by arterial blood sampling.

**Methods:**

To segment the trunk of the major arteries in the neck, a high-resolution time-of-flight MRI angiography was postprocessed by a vessel-enhancement filter based on the inertia tensor. Together with the measured PSF of the PET subsystem, the arterial mask was used for geometrical deconvolution, yielding the time-resolved activity concentration averaged over a major artery. The method was compared to manual arterial blood sampling at the hind leg of 21 sheep (animal stroke model) during measurement of blood flow with O15-water. Absolute quantification of activity concentration was compared after bolus passage during steady state, i.e., between 2.5- and 5-min post injection. Cerebral blood flow (CBF) values from blood sampling and IDIF were also compared.

**Results:**

The cross-calibration factor obtained by comparing activity concentrations in blood samples and IDIF during steady state is 0.98 ± 0.10. In all examinations, the IDIF provided a much earlier and sharper bolus peak than in the time course of activity concentration obtained by arterial blood sampling. CBF using the IDIF was 22 % higher than CBF obtained by using the AIF yielded by blood sampling.

**Conclusions:**

The small deviation between arterial blood sampling and IDIF during steady state indicates that correction of PVE and PSF is possible with the method presented. The differences in bolus dynamics and, hence, CBF values can be explained by the different sampling locations (hind leg vs. major neck arteries) with differences in delay/dispersion. It will be the topic of further work to test the method on humans with the perspective of replacing invasive blood sampling by an IDIF using simultaneous PET/MRI.

## Background

To quantify metabolic or physiological function by means of of radiotracers in positron emission tomography (PET), kinetic models are applied to calculate the rate constants between different compartments, e.g., between blood and tissue. For these models, the measurement of the arterial input function (AIF) is mandatory. The AIF is the time-activity concentration curve of an injected radiopharmaceutical in arterial blood plasma that is delivered to the target tissue. This activity curve is normally determined by taking arterial blood samples from the patient at different time points after injection.

As a potential alternative, the AIF could also be obtained from the PET data itself, yielding an image-derived input function (IDIF). Different approaches have been taken to calculate the IDIF. Firstly, the IDIF can be derived from a blood pool (major vessels, ventricles of the heart) visible in the low-resolution PET data using mathematical modeling and/or a few blood samples for calibration [1–11]. Secondly, data from a co-registered high-resolution imaging technique can be used to segment the arterial blood pool. These can be magnetic resonance imaging (MRI) [12–15] as well as computed tomography (CT) images [16].

To obtain an accurate estimation of IDIF, the so-called spill-in and spill-out, partial-volume effects (PVE) have to be corrected for. For this correction, the exact knowledge about the point spread function (PSF) response of the PET imaging system and the geometry (diameter, orientation) of the artery is necessary. However, the co-registration of structures in sequentially acquired sessions in different imaging modalities may be difficult because, e.g., the carotid artery changes its exact position with the head position and global registration algorithms usually are based on other structures. Using hybrid PET/MRI scanners where the images can be acquired simultaneously allows for a precise segmentation of the artery [14].

To be useful in research and clinical practice, manual operation has to be minimized while generating the IDIF. Ideally, the IDIF is generated fully automated from the simultaneous PET/MRI data. This work describes such a procedure applied to a study with an animal model (sheep). It compares the generated IDIF with an AIF obtained by blood sampling to quantify cerebral blood flow (CBF) by ^15^O-H_2_O-PET imaging. Moreover, CBF values obtained by both methods are compared.

## Methods

If not mentioned otherwise, the object-oriented development interface for NMR [17] (URL: http://od1n.sourceforge.net/) was used for all data processing steps.

### Animal model and imaging procedure

The animal experiments were conducted in accordance with the recommendations of the European Convention for the Protection of Vertebrate Animals used for Experimentation and the current ARRIVE guidelines. The animal experiments were approved by the local animal welfare authorities (Directorate Leipzig, Germany). The examinations were part of a preclinical stroke study, investigating therapeutic effects of stem cell application. For that purpose, adult merino sheep (*n*=21; 62 ± 8-kg body weight) underwent permanent proximal middle cerebral artery occlusion as described previously [18, 19]. Examinations were performed on a clinical PET/MRI scanner (Biograph mMR, Siemens Healthcare, Erlangen, Germany) after induction of ischemia (from 4 h to 35 days after stroke). MR signal was received by a flexible body surface coil wrapped over the animal head in addition to the spine coil which is integrated in the patient table. The surface coil is not accounted for in the attenuation correction. Imaging included structural (e.g., T1-weighted, diffusion-tensor imaging), diagnostic (e.g., fluid-attenuated inversion recovery, turbo spin echo), and functional (dynamic susceptibility contrast-based perfusion imaging, arterial spin labeling) MRI sequences.

A time-of-flight MRI angiography (TOF-MRA) sequence was used to identify the trunk of the major neck arteries. Parameters of the sequence were as follows: 0.5 × 0.5 × 0.7 mm ^3^ voxel size, 4 slabs, 40 slices per slab, TE = 3.6 ms, TR = 21 ms, 21° flip angle, GRAPPA = 2, saturation pulse for venous signal, and 70 % tilted optimized nonsaturating excitation pulse.

To determine CBF, sheep were subjected to a 5-min ^15^O-H_2_O-PET scan starting with a bolus injection of 1064 ± 238 MBq (mean and standard deviation over subjects) into the jugular vein. Blood samples were withdrawn manually from a femoral artery (left/right side depending on accessibility of arterial cannulation) during the PET scan. For the first 2 min after tracer injection, blood sampling was performed dynamically (approximately every 3 s), followed by predefined time points: 150, 180, 210, 240, and 300 s. Recording the sampling procedure with a video camera allowed for temporal synchronization to the start of PET scan as well as a retrospective definition of the exact sampling time points. The blood activity concentration was measured using a gamma counter (WIZARD2, PerkinElmer LAS GmbH, Rodgau, Germany) cross-calibrated to the PET/MRI scanner. After an upgrade of the blood sampling procedure, the simultaneous TOF-MRA/PET measurements (see below) were performed by an automated blood sampler (Twilite, Swisstrace GmbH, Menzingen, Switzerland) also cross-calibrated to the PET/MRI.

PET data was reconstructed into a 128 × 128 matrix (voxel size: 1.40 × 1.40 × 2.03 mm^3^) using the built-in 3D ordered subset expectation maximization (OSEM) algorithm with 8 iterations, 21 subsets, and a 3-mm Gaussian filter. Scatter and Dixon-based attenuation correction were applied. The following consecutive time frames were used for the dynamic analysis: 20 × 3, 12 × 5, 12 × 10, and 2 × 30 s.

### Segmentation of major arteries

**Fig. 1:**
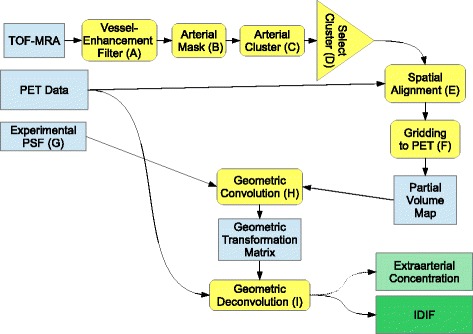
Flowchart outlining the whole procedure to derive the IDIF. Datasets and intermediate results are coded in *blue*, processing steps in *yellow*, and final results in *green*. *Capital letters* are referenced in the text

In a first step in generating IDIFs, the main arteries in the sheep neck supplying the brain were identified. Although dedicated MR angiography methods exist, which highlight arterial blood, and hence, vessels, segmentation based on the contrast of these sequences alone can be problematic due to artifacts (e.g., due to inhomogeneous coil profiles leading to large-scale intensity variations). Hence, vessel-enhancement filtering is a mandatory step (step A in Fig. 1). This filter consists of an image transformation to enhance the main feature of blood vessels, namely their tubular shape. In the present approach, the filter/transformation was based on the inertia matrix within a predefined sphere [20, 21]. The initial purpose of this approach was to identify center lines of vessels. In the context of IDIF generation, we modified this approach to differentiate between arterial and other voxels of the TOF-MRA.

**Fig. 2:**
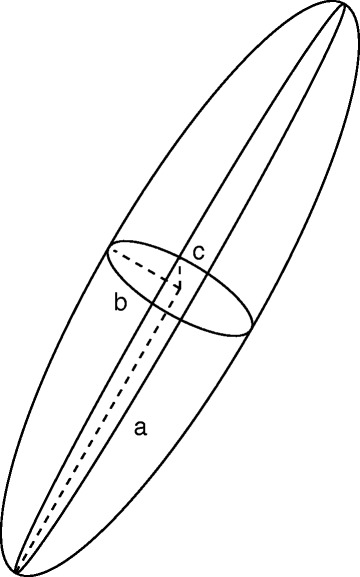
Ellipsoid representing the inertia tensor used in the vessel-enhancement filter. For prolate shapes of the ellipsoid, corresponding to vessel-like structures in the TOF-MRA data, one of the principal radii, *a*, *b*, *c*, will be larger than the other two

For each voxel, a sphere of a certain radius was defined around this voxel. Using all voxels inside the sphere, the inertia tensor centered on the gravity center of the sphere was calculated by interpreting voxel intensity values and positions as discrete masses. The eigenvalues of the tensor, *λ*_1_≤*λ*_2_≤*λ*_3_, are related to the principal radii, *a*, *b*, *c* of the corresponding inertia ellipsoid (Fig. 2):

(1) $$ a=\frac{1}{2\sqrt{\lambda_{1}}}, \quad b=\frac{1}{2\sqrt{\lambda_{2}}}, \quad c=\frac{1}{2\sqrt{\lambda_{3}}}.  $$

If the ellipsoid has a prolate shape (cigar), one of the radii (e.g., *a*) will be much larger than the other two radii. Consequently, one of the eigenvalues (e.g., *λ*_1_) will be much closer to zero than the other two eigenvalues:

(2) $$ \lambda_{1} \approx 0, \quad \lambda_{2}\approx\lambda_{3} \gg 0.  $$

Hence, the difference *λ*_2_−*λ*_1_ will be large for prolate shapes. To obtain a scale-invariant measure of “prolateness,” *p*, this difference was normalized by the sum of eigenvalues:

(3) $$ p=\frac{\lambda_{2}-\lambda_{1}}{\lambda_{1}+\lambda_{2}+\lambda_{3}}.  $$

**Fig. 3:**
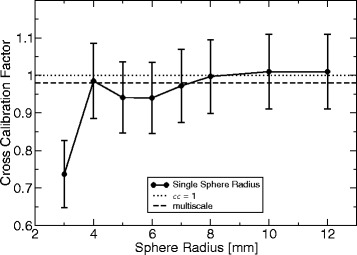
Cross-calibration factor for different sphere radii of the inertia-based vessel-enhancement filter. The cross-calibration factor (Eq. 7) based on the PET-aligned consecutive TOF-MRA data was calculated using different single sphere radii. Smaller radii did not allow an automated vessel segmentation and therefore, yielded no value for *cc*. The result of the multiscale combination (Eq. 4) is also shown

The sensitivity of *p* to vessels of certain size will depend on the radius, *r*, of the sphere used to calculate *p*, i.e., by using a large sphere, *p* will be sensitive to larger vessels. To obtain a homogenous sensitivity for a wide range of vessel sizes and to avoid picking a certain arbitrary radius which would be optimal for the current study, but might lead to suboptimal results in another study (humans), *p* is calculated for different *r* and the results were combined for each voxel. To reduce noise in this multiscale combination, the sum of squares was used for the combination, i.e., the final prolateness *P* was calculated voxel-wise by

(4) $$ P=\sqrt{\sum\limits_{i=1}^{N} \left[ p(r_{i}) \right]^{2}}  $$

with *r*_*i*_ as the radius of the *i*th sphere and *N* as the total number of spheres. The minimum useful radius corresponds to two voxels, whereas the impact of the maximum radius is small (cf. Fig. 3) but should not be chosen too large, i.e., on a completely different spatial scale compared to the typical diameter of an artery (6–8 mm). Otherwise, other large-scale tubular structures, such as the animals neck, could be amplified by the filter. Moreover, the computational expense to calculate the inertia tensor increases with *r* and *N*. In this work, a maximum sphere radius of 8 mm was used. For comparison, *P* (*N*=1 and hence, *P*=*p*) was also calculated for single discrete radii in the range of 3–12 mm. It was then used for the same analysis (described below) as the multiscale-filtered data. In the last step, the TOF-MRA data was multiplied voxel-wise by *P* for vessel enhancement. To summarize, the above analysis of the geometrical shape based on the vicinity (surrounding voxels) is calculated for each voxel by Eq. 4 to yield a degree of resemblance to vessel-like structures. This filter is applied voxel-wise to enhance vessel-like structures in the TOF-MRA data.

**Fig. 4:**
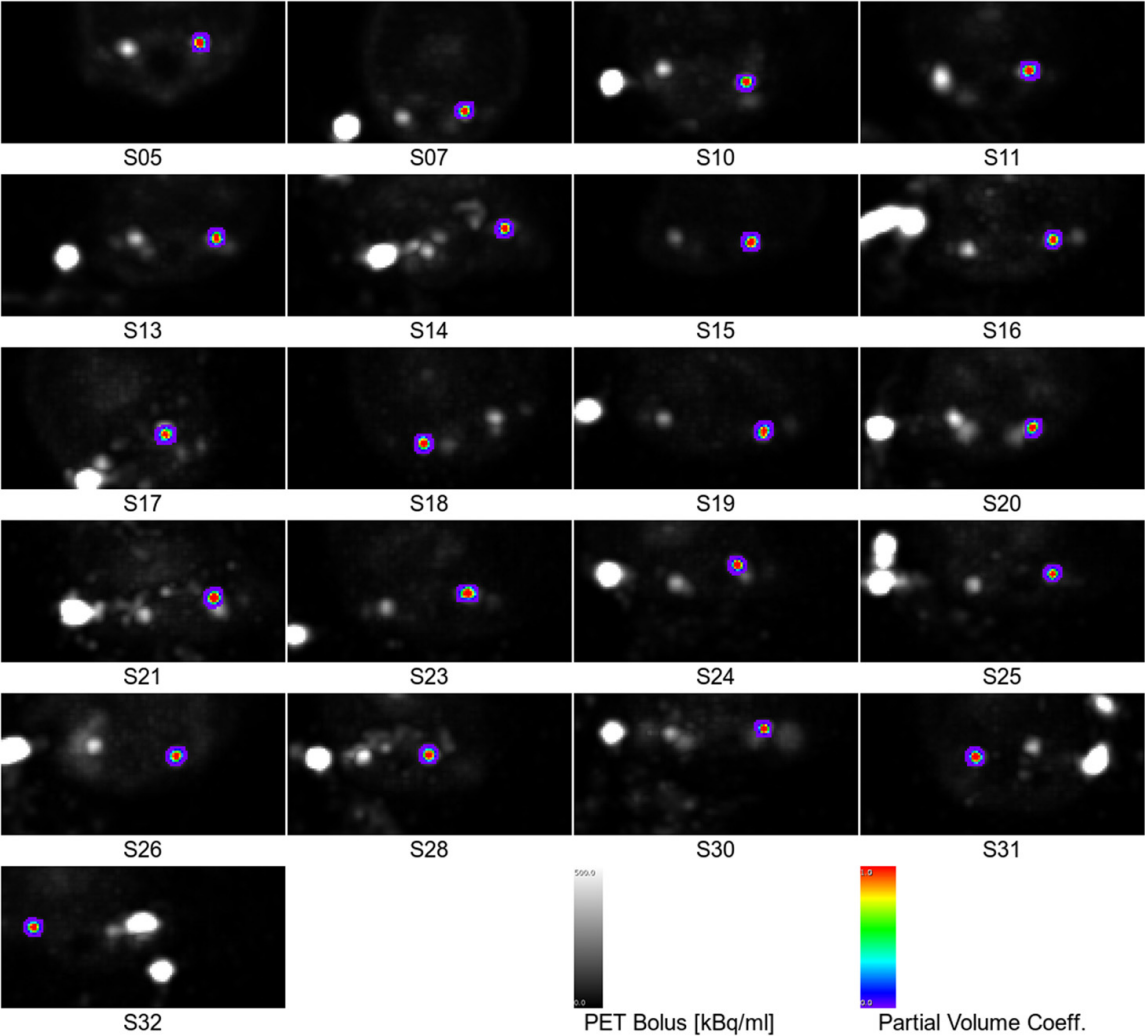
Arterial segmentations from MR angiography overlaid onto transverse cross-sections of the PET data. For visibility, the maximum peak intensity over the whole PET time course is used, i.e., maximum intensity projection is performed over the time dimension

**Fig. 5:**
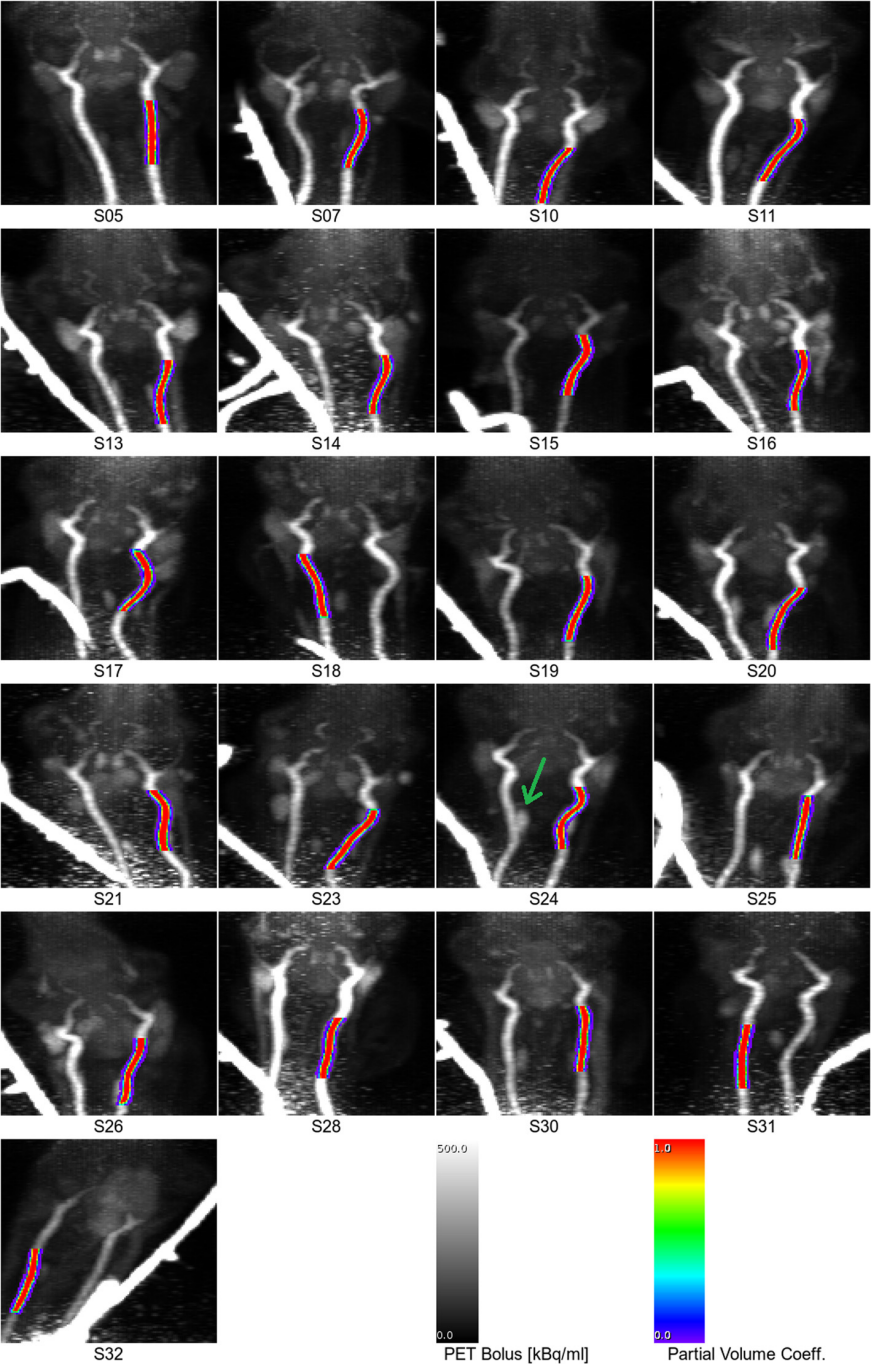
Arterial segmentations from MR angiography overlaid onto coronal maximum-intensity projections of the PET data. As in Fig. 4, the maximum peak intensity over the whole PET time course is used, i.e., maximum intensity projection is performed over two dimensions: the anterior-posterior direction and the time dimension. The capillary tube (catheter) which was used for injection of ^15^O-water is also visible. The *green arrow* in S24 indicates the rete mirabile (see text)

After applying the vessel-enhancement filter described above, the following procedure was used to segment the arteries in the enhanced TOF-MRA data by defining a mask containing only arterial voxels (step B in Fig. 1). For that, the threshold of the image intensity to outline the mask was determined from a histogram of image intensities (100 bins). The threshold was set to the first minimum above zero in this histogram so that the regions with low image intensity (air, soft tissue, and bone) are excluded. This step is necessary since TOF-MRA signal intensities do not relate directly to a physical quantity to which a simple fixed threshold could be applied. The resulting masks gave good outlines of the arteries, as verified by visual inspection (cf. Figs. 4 and 5).

To segment only the major arteries, clusters of adjacent voxels are generated (step C in Fig. 1). The two largest clusters, which correspond to the straight parts of the bilateral carotid arteries, are selected automatically (step D in Fig. 1). Finally, to avoid bias from the extended peripheral venous catheter which was used for injection of ^15^O-water (Fig. 5), only the artery on the contralateral side was used, i.e., furthest from the catheter. This artery was selected automatically based on the maximum distance of its center of gravity (COG) (arterial mask interpreted as discrete masses) from the PET COG (maximum PET bolus activity concentration interpreted as discrete masses).

### Spatial alignment

Although a simultaneous PET-MRI system was used which provides inherent co-registration, the TOF-MRA data for segmentation of arteries was acquired approximately 20 to 30 min prior to the PET acquisition. This offset was dictated by other constrains of the overall study.

Although animals were anesthetized, we observed a spatial misalignment between TOF-MRA and PET, probably caused by slow sinking/rolling of the animal during this period of time. This misalignment was corrected for by the following procedure (step E in Fig. 1). Because the principal direction of the arteries is in axial direction, it was assumed that the major impact on results is caused by motion perpendicular to the axial direction. Thus, motion correction was performed slice-by-slice in a two-dimensional fashion. In each transaxial slice, the COG of the segmented arterial voxels was compared with the position of the maximum of PET signal. This two-dimensional shift was fitted as a function of slice position to a second-order polynomial to obtain a smooth slice-to-slice transition. Finally, the fitted shift was used in a slice-by-slice spline interpolation to align the artery segmentation (binary mask) to the PET images (step F in Fig. 1). As described previously [22], this procedure yielded a partial-volume coefficient (values between 0 and 1) assigned to each PET voxel which reflects the fractional overlap with the TOF-MRA mask.

To test whether the alignment procedure would be necessary in a truly simultaneous TOF-MRA and PET examination, 4 sheep were investigated with the TOF-MRA sequence acquired during the PET scan.

### Partial-volume effect and point-spread correction

Having identified and aligned the arteries with high resolution, the next step is to compensate for spill-over effects in the low-resolution PET data. For that, the PSF has to be known. To obtain the PSF of the PET imaging modality (detector hardware and reconstruction algorithm), the following calibration experiment was performed once (G in Fig. 1): a 1-ml syringe with a known diameter of 4.7 mm (cylinder length: 57 mm) was filled with 95-MBq ^18^F-fludeoxyglucose. The syringe was placed axially in the scanner in approximately the same off-center position (50 mm) where the TOF-MRA volume would be in the actual ^15^O-H_2_O-PET/MRI examination. PET data was acquired and reconstructed in the same way as in the actual examinations. For 16 axial slices, which were perpendicular to the axis of the syringe and included a cross-sectional image in the central part of the syringe (i.e., without edge effects), the following procedure was applied to estimate the full-width at half-maximum (FWHM) of the PSF. A model function was fitted to each slice using a downhill simplex algorithm. The function was composed of a disk shape (syringe cross-section, i.e., 1 inside the syringe and 0 outside) convolved with a Gaussian (representing the PSF) and allowed a variable position inside the imaging plane. Four independent parameters of the function were used in the fitting procedure: the two-dimensional position of the disk, the amplitude, and the FWHM of the Gaussian. Averaging over all slices yielded an average FWHM.

The data from the PSF experiment was also used to estimate the bias in activity concentration as a function of different frame durations which might be introduced by the OSEM reconstruction [23]. For this estimation, a mask containing only the syringe was generated by the same histogram-based algorithm as used for segmentation of arteries (see above). For each time frame, activity was averaged over all voxels contained in the mask. Finally, it was normalized to the mean over time.

Convolving the partial-volume map (as obtained by step F in Fig. 1) with the PSF yielded a partial-volume coefficient per voxel which also takes PSF effects (spill-in and spill-out) into account (step H in Fig. 1). Using this coefficient, the activity concentration of all voxels in a predefined region (all voxels not further away than two FWHM from the next arterial voxel) was modeled by a linear combination of intra- and extraarterial concentration. This geometric transformation matrix [24], which is a massively overdetermined set of linear equations, was inverted and applied to each time frame (step I in Fig. 1), yielding the IDIF and the extraarterial activity concentration.

### Comparison of IDIF and AIF

As different sampling locations are used for IDIF and the blood-sample-based AIF (hind leg vs. major neck arteries) with differences in delay/dispersion, differences in bolus dynamics can be expected. Hence, to compare the IDIF and the blood-sample-based AIF free of bolus dynamics, it was assumed that in the second half of the 5-min PET acquisition, the distribution of the tracer in the arterial blood reaches a homogenous steady state, i.e., the concentration in arteries of the neck (IDIF) is the same as in the location of drawing the blood samples (AIF). The mean and standard deviation of the IDIF frames, $\overline {\textrm {IDIF}}_{i}$ and *σ*(IDIF)_*i*_, and AIF blood samples, $\overline {\textrm {AIF}}_{i}$ and *σ*(AIF)_*i*_, were calculated for the *i*th subject during this period. The subject-specific cross-calibration factor, *cc*_*i*_, which presents the ratio of AIF to IDIF for a single subject, was obtained by

(5) $$ {cc}_{i}=\overline{\textrm{AIF}}_{i}/\overline{\textrm{IDIF}}_{i}\,.  $$

The standard deviation was used to take the reliability of each *cc*_*i*_ into account in the following way: a high fluctuation (large standard deviation) of measured activity concentration in the blood samples and/or IDIF of one subject was considered to be associated with a low reliability of the corresponding *cc*_*i*_, and vice versa. Quantitatively, and after removing any remaining linear trend over time, this interrelationship can be employed by analysis of error propagation and calculating the standard error/deviation of *cc*_*i*_:

(6) $$ \sigma({cc}_{i})=\sigma(\textrm{AIF})_{i}\frac{1}{\overline{\textrm{IDIF}}}_{i}+\sigma(\textrm{IDIF})_{i} \frac{\overline{\textrm{AIF}}_{i}}{\overline{\textrm{IDIF}}_{i}^{2}}  $$

The global cross-calibration factor, *cc*, between IDIF and blood samples was obtained by the reliability-weighted average over *n* subjects,

(7) $$ cc=\frac{\sum_{i=1}^{n} w_{i} \cdot {cc}_{i}}{\sum_{i=1}^{n} w_{i}}\,  $$

with *w*_*i*_=1/*σ*(*cc*_*i*_) taken as the reliability of *cc*_*i*_ of the *i*th subject. This factor can be either used to compare AIF and IDIF as done in this study or to cross-calibrate both (not applied in this study). Finally, the analysis of error propagation yielded the standard error of *cc*:

(8) $$ \sigma(cc)=\frac{\sum_{i=1}^{n} w_{i} \cdot \sigma({cc}_{i})}{\sum_{i=1}^{n} w_{i}}\, =\frac{n}{\sum_{i=1}^{n} 1/\sigma({cc}_{i})}.  $$

In addition to the cross-calibration factor, the ratio of intra- vs. extraarterial (tissue surrounding vessel) activity concentration, as obtained by the geometrical deconvolution described above, was evaluated in steady state. Furthermore, the area under curve (AUC) of IDIF and AIF was compared for the whole scan (0–300 s).

CBF maps were calculated using either AIF or IDIF for kinetic modeling with PMOD software (PMOD Technologies Ltd, Zurich, Switzerland) applying the weighted integration method according to Alpert et al. [25] using the whole 5-min scan. In case of AIFs, where the blood tracer activity concentration is recorded with an external device, the measured activity concentration is distorted relative to the activity signal arriving in the brain by two effects: a tracer time delay and a broadening of the peak of the AIF (bolus dispersion). For exact CBF measurements, the delay and dispersion of the blood measurements must, therefore, be corrected for. In our study, the PMOD-implemented method by Meyer [26] was used. CBF values were averaged over a manually drawn region covering the healthy hemisphere (i.e., not directly affected by stroke) for each subject. In the group with simultaneous TOF-MRA and PET, one sheep had to be excluded from the CBF analysis because of missing arterial blood data during bolus passage.

## Results

**Fig. 6:**
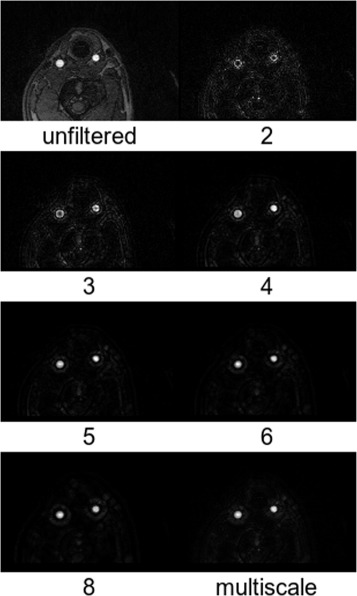
Transverse cross-sections of the vessel-filtered TOF-MRA data as a function of sphere radius in mm. The original unfiltered TOF-MRA data and the result from the multiscale combination (Eq. 4) is also shown

**Fig. 7:**
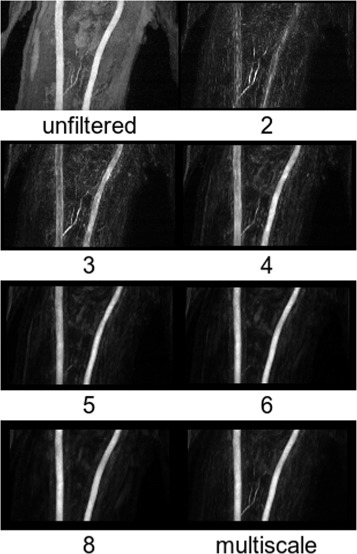
Coronal maximum intensity projections of the vessel-filtered TOF-MRA data as a function of sphere radius in millimeters. The original unfiltered TOF-MRA data and the result from the multiscale combination (Eq. 4) is also shown

Examples of the effect of the vessel enhancement filter are shown in Figs. 6 and 7. Compared to the unfiltered data, robust delineation of arteries could be obtained by the multiscale combination (Eq. 4). Figure 3 shows the cross-calibration factor (Eq. 7) if different single vessel radii are used. The results are very close to identity for all radii above a certain sphere radius (4 mm).

**Fig. 8:**
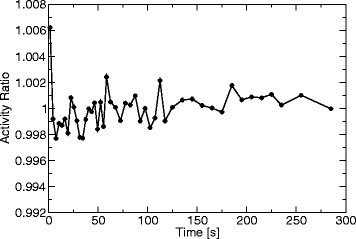
Variation of activity over time in the measurement for PSF evaluation. Values are normalized to the mean over the whole time course

For the PET reconstruction used in this study, the FWHM of the PSF when approximated by a Gaussian function is 6.86 ± 0.01 mm (mean and standard deviation over slices). The small standard deviation indicates a reproducible and robust modeling/fitting of the PSF. The same experiment yielded an estimate of the bias over time, i.e., as a function of frame duration (Fig. 8). Except for the first frame, the bias is on the order of 0.1 %.

The transaxial spatial shift in the alignment procedure, averaged over all slices, was 3.18 ± 1.69 mm (mean and standard deviation over subjects) with a maximum of 8.94 mm for the consecutive TOF-MRA/PET. For the simultaneous TOF-MRA/PET, it was 2.02 ± 0.57 mm with a maximum of 2.66 mm.

The segmented arteries of all sheep from the consecutive TOF-MRA and PET examinations are shown in Figs. 4 and 5. Visually, good segmentation of arteries can be observed. The algorithm reliably detects the artery on the opposite side of the catheter to reduce bias from the catheter.

**Fig. 9:**
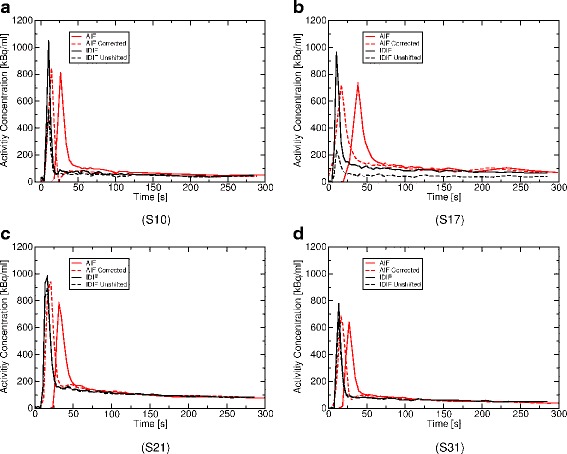
Example AIFs and IDIFs from four sheep (**a**–**d**). The delay- and dispersion-corrected AIFs (AIF corrected) used for CBF calculation are shown in addition to the uncorrected AIFs

Regarding the example AIFs and IDIFs in Fig. 9, two observations can be made: Firstly, the IDIF provides an earlier and narrower bolus peak than the blood samples. Secondly, spatial alignment in the consecutive TOF-MRA and PET leads to a higher peak in the IDIFs and makes AIF and IDIF become comparable in steady state (between 2.5- and 5-min post injection).

**Fig. 10:**
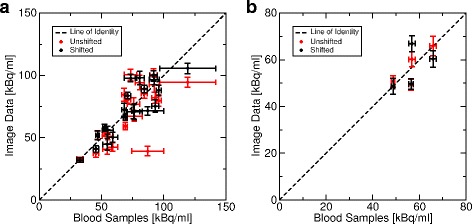
Arterial ^15^O-H_2_O activity concentrations in steady state (between 2.5- and 5-min post injection) as measured by blood sampling and IDIF. Each point represents one subject. The standard deviations as used in Eq. 6 are shown as error bars. **a** Data from the non-simultaneous (i.e., consecutive) TOF-MRA and PET measurements, whereas **b** The results from simultaneous TOF-MRA and PET. The data labeled ‘Shifted’ stems from the PET-aligned (i.e., motion-corrected) TOF-MRA data

The comparison of AIF (blood samples) vs. IDIF in steady state is shown in Fig. 10. Standard deviations of AIF values are generally higher than those of IDIF. The corresponding cross-calibration factors are summarized in Table 1. The coefficients are not significantly different from identity (two-tailed, one sample *t*-test, *p* < 0.05).

**Table 1:** Parameters of IDIF and AIF for consecutive and simultaneous TOF-MRA/PET acquisition

	Shifted			Unshifted		
Group	*cc*	IA/EA	AUC _IDIF_/AUC _AIF_	*cc*	IA/EA	AUC _IDIF_/AUC _AIF_
Consecutive	0.98 ±0.10	3.17 ±0.66	0.93 ±0.09	1.01 ±0.11	2.89 ±0.72	0.84 ±0.17
Simultaneous	1.01 ±0.08	2.78 ±0.96	0.96 ±0.13	1.01 ±0.07	2.76 ±0.88	0.91 ±0.06

The cross-calibration factor, *cc*, is given in the form *c* *c*±*σ*(*c* *c*) (cf. Eqs. 7 and 8). The value IA/EA represents the ratio of activity concentration between the intraarterial and the extraarterial space as mean and standard deviation over subjects in steady state. The data labeled ‘Shifted’ stems from the PET-aligned TOF-MRA data, whereas ‘Unshifted’ labels results from the non-aligned data

According to Table 1, the intraarterial activity concentration in steady state is approximately three times higher than in the extraarterial space. It is worth noting that this factor is significantly increased by the spatial alignment for the consecutive TOF-MRA/PET data, while it remains almost the same for the simultaneous TOF-MRA/PET. Taking the consecutive and simultaneous TOF-MRA/PET together, the AUC of the AIF is approximately 5 % higher than the AUC of the IDIF for the spatially aligned data (labeled ‘Shifted’ in Table 1).

**Fig. 11:**
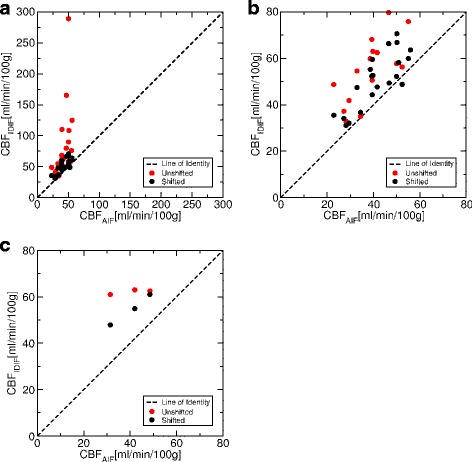
Comparison of CBF derived using either blood sampling (AIF) or IDIF. Each point represents one subject. **a** Data from the non-simultaneous (i.e., consecutive) TOF-MRA and PET measurements. **b** The same data within a smaller range of CBF values (zoom). **c** The data from simultaneous TOF-MRA and PET. The data labeled ‘Shifted’ stems from the PET-aligned TOF-MRA data

Figure 11 compares CBF values obtained by AIF and IDIF, and Table 2 summarizes the quantitative results. On average, CBF of IDIF is 22 % higher than CBF obtained by blood sampling. This difference is significant (two-tailed, one sample *t* test, *p* < 0.05). Hence, a systematic deviation can be assumed when comparing CBF of IDIF and AIF.

**Table 2:** CBF values obtained by IDIF and AIF for consecutive and simultaneous TOF-MRA/PET acquisition

	Shifted	Unshifted	
Group	CBF _IDIF_	CBF _IDIF_	CBF _AIF_
Consecutive	50.7 ±12.0	81.5 ±58.0	41.5 ±9.7
Simultaneous	54.6 ±6.6	62.2 ±1.1	40.6 ±8.6

Values are given in ml/min/100g as mean and standard deviation over subjects. The data labeled ‘Shifted’ stems from the PET-aligned TOF-MRA data, whereas ‘Unshifted’ labels results from the non-aligned data

Comparing the aligned vs. the unaligned data, a major improvement can be seen for the consecutive TOF-MRA/PET data by aligning the TOF-MRA data, i.e., the standard deviation of IDIF-based CBF is reduced by a factor of 5 when the TOF-MRA data is shifted to match spatial position of the arteries in PET. This improvement could not be seen in the simultaneous TOF-MRA/PET. Also, CBF values of simultaneous TOF-MRA/PET are more similar than for the consecutive TOF-MRA/PET. However, the extremely small standard deviation of the unshifted IDIF-based CBF of the simultaneous TOF-MRA/PET in Table 2 is most likely only a coincidence and not representative due to the small sample size (*n*=3). Hence, it is difficult to say whether a significant difference (e.g., as estimated by a *t* test) remains between simultaneous TOF-MRA/PET and motion-corrected consecutive TOF-MRA/PET.

## Discussion

Our results show that the combination of TOF-MRA, vessel-enhancement filter, and automated mask selection allows a robust segmentation of arteries. Another approach to enhance vessel-like structures based on the second-order directional derivatives (Hessian matrix) [27] might also be feasible instead of evaluating the inertia tensor. Interactive selection of a 3D seed point, as for instance in [20], is not required for the approach presented here. Thus, the present method allows a fully automated calculation of IDIF shortly after the data was acquired, allowing, for instance, rapid calculation of CBF maps. This is of importance for examinations where timely diagnosis is mandatory, e.g., in stroke.

There is good agreement in activity concentrations of blood samples and IDIF after bolus passage during steady state. Conversely, CBF values are significantly higher when based on IDIF. The most plausible explanation for this deviation are the different sampling locations (hind leg vs. major neck arteries) with differences in delay/dispersion (including their correction by PMOD in case of AIF) leading to different bolus dynamics.

One may argue that the good agreement in steady state is simply a consequence of a homogenous distribution of the tracer in the body far after bolus passage due to perfusion and extravasation of ^15^O-H_2_O into the extrarterial space, and that the differences in CBF (approximately 20 %) arise from an insufficient quantification of tracer concentration during bolus passage. However, as tissue CBF is inversely proportional to the arterial AUC, this effect may only account for approximately 5 % of the difference. Moreover, a three-times higher activity concentration in the intraarterial space compared to extraarterial space in steady state (cf. Table 1) indicates that the tracer is not homogenously distributed in steady state after bolus passage.

The measured activity concentration is relatively independent of the frame duration (cf. Fig. 8). Only the first frame shows a considerable bias. However, as the IDIF is negligibly small in the first frame, the impact on CBF analysis will be small.

Another source of quantification error could be an insufficient attenuation correction as the Dixon-based method does not account for bones. Moreover, the flexible surface coil is not accounted for in the attenuation correction. However, the good agreement in activity concentrations of blood samples and IDIF indicates that these sources of error have a minor impact on quantification. This might be because there are fewer bones in the neck compared to the brain/head. Also, the surface coil was distant from the neck.

The comparison of consecutive (i.e., with a delay of 20 to 30 min) with the simultaneous TOF-MRA/PET suggests that retrospective spatial alignment is a mandatory step in case that angiography and PET cannot be acquired truly simultaneously, e.g., when dictated by other constrains of the overall study, as in this work. Our data suggest that spatial registration is not necessary in simultaneous TOF-MRA/PET. However, a residual difference in CBF values before and after spatial alignment can be observed even in the simultaneous TOF-MRA/PET data. This difference can be explained by other effects. For instance, field distortions can cause spatial displacements in MRI. Also, the bolus passage at the beginning of the 5-min scan is the crucial time for the CBF analysis, whereas TOF-MRA is acquired slab-by-slab during the whole scan. Thus, subject motion during the scan can also cause differences in CBF.

An additional problem that may arise if the input function is calculated only from image data is the fact that the initial rapid rise and decay of the input function curve cannot be sampled very precisely from the PET data due to its low temporal resolution. On the other hand, by using a PET/MRI system with a relatively large axial PET FOV (258 mm) in this study, a large arterial volume is available within the FOV for the calculation of the IDIF. Therefore, more radioactive decays are present in the arterial volume under observation, and the temporal resolution of the IDIF can possibly be increased while maintaining a reasonable number of counts per sampling interval in the IDIF.

Unlike humans, sheep have an arterial structure (*rete mirabile*) consisting of a complex net of arteries close to the major neck arteries. This structure is visible in the early PET images, for instance, in S24 in Fig. 5 (indicated by a green arrow). In contrast, this structure is not visible in the TOF-MRA data. This is most likely because the TOF-MRA method is only sensitive to blood flowing into the imaging slab with a relatively high velocity. Therefore, the blood in this structure may bias quantification by uncorrected spill-over effects in PET. However, the relatively good agreement of blood samples and IDIF in steady state suggests that this structure is not a major source of error in the present study.

It should be straightforward to extend the present method to humans, provided that segmentation of the arteries is adapted. This may include fine tuning the vessel-enhancement filter (maximum sphere radius) and extending the number of arterial clusters to four (internal carotid arteries). Also, it should be possible to apply the method to studies with other radiotracers. Depending on the tracer, additional blood samples might be neccessary to determine the plasma input function. If long dynamic PET scans are used (e.g., ^18^F-fludeoxyglucose (FDG) uptake during approximately 1 h), it would be useful to repeat the TOF-MRA measurement during this time in order to track potential movement of the arteries.

## Conclusions

The small deviation between arterial blood sampling and IDIF during steady state indicates that the correction of PVE and PSF is possible with the method presented. The differences in bolus dynamics and, hence, CBF values can be explained by the different sampling locations (hind leg vs. major neck arteries) with differences in delay/dispersion. It will be the topic of further work to test the method on humans with the perspective of replacing invasive blood sampling by an IDIF using simultaneous PET/MRI.
